# Inactivation of Cerebellar Cortical Crus II Disrupts Temporal Processing of Absolute Timing but not Relative Timing in Voluntary Movements

**DOI:** 10.3389/fnsys.2016.00016

**Published:** 2016-02-24

**Authors:** Kenji Yamaguchi, Yoshio Sakurai

**Affiliations:** ^1^Department of Psychology, Graduate School of Letters, Kyoto UniversityKyoto, Japan; ^2^Japan Society for the Promotion of ScienceTokyo, Japan; ^3^Laboratory of Neural Information, Graduate School of Brain Science, Doshisha UniversityKyotanabe, Japan

**Keywords:** temporal processing, cerebellum, pharmacological inactivation, voluntary movement, rats

## Abstract

Several recent studies have demonstrated that the cerebellum plays an important role in temporal processing at the scale of milliseconds. However, it is not clear whether intrinsic cerebellar function involves the temporal processing of discrete or continuous events. Temporal processing during discrete events functions by counting absolute time like a stopwatch, while during continuous events it measures events at intervals. During the temporal processing of continuous events, animals might respond to rhythmic timing of sequential responses rather than to the absolute durations of intervals. Here, we tested the contribution of the cerebellar cortex to temporal processing of absolute and relative timings in voluntary movements. We injected muscimol and baclofen to a part of the cerebellar cortex of rats. We then tested the accuracy of their absolute or relative timing prediction using two timing tasks requiring almost identical reaching movements. Inactivation of the cerebellar cortex disrupted accurate temporal prediction in the absolute timing task. The rats formed two groups based on the changes to their timing accuracy following one of two distinct patterns which can be described as longer or shorter declines in the accuracy of learned intervals. However, a part of the cerebellar cortical inactivation did not affect the rats’ performance of relative timing tasks. We concluded that a part of the cerebellar cortex, Crus II, contributes to the accurate temporal prediction of absolute timing and that the entire cerebellar cortex may be unnecessary in cases in which accurately knowing the absolute duration of an interval is not required for temporal prediction.

## Introduction

Research on the cerebellum has in recent years focused on the cognitive functions, although until the beginning of the 1990s the role of the cerebellum was thought to be solely the control of motor functions (Ito, 2008). It has also been suggested that the cerebellum plays an important role in rapid temporal processing (on the scale of milliseconds) than in any other cognitive function (Galliano et al., 2013; Rahmati et al., 2014). Several studies on brain damage (Ivry and Keele, 1989; Keele and Ivry, 1990); brain imaging (Rao et al., 2001; Dreher and Grafman, 2002; Sakai et al., 2002; Lewis and Miall, 2003; Xu et al., 2006), and brain stimulation (Koch et al., 2007; Grube et al., 2010; and reviewed in Tomlinson et al., 2013) involving human subjects have demonstrated that subsecond temporal processing is deeply associated with the cerebellum. Similarly, studies involving animals (Kotani et al., 2003; Jirenhed et al., 2007; Svensson et al., 2010; Jirenhed and Hesslow, 2011a,b; Ohmae et al., 2013; Wetmore et al., 2014) have reported specific spiking activity patterns of the cerebellum while the animals were predicting events that happened subseconds later.

The question of whether the cerebellar timing function is involved in the temporal processing of discrete or continuous events has been controversial (Ivry et al., 2002; Yamaguchi and Sakurai, 2014b). Temporal processing during discrete events functions by counting absolute time like a stopwatch. In contrast, during continuous events it works by measuring events at intervals. During the temporal processing of continuous events, animals might respond to the rhythmic timing of sequential responses rather than to the absolute duration of intervals between events. A brain stimulation study (Grube et al., 2010) has reported that the cerebellum was only involved in the temporal processing of the absolute time at which discrete events occurred. On the other hand, other research on impairment (Bo et al., 2008) and electrophysiology (Ohmae et al., 2013) has suggested that the cerebellum is important for both absolute and continuous (relative) timings. Research that compares behavioral performance under partial inactivation of the cerebellum during absolute- and relative-timing tasks, without any change in movement, is needed to address these questions.

Here we investigate the contribution of the cerebellar cortex to the temporal processing of absolute and relative timing, using voluntary movement tasks. In a previous study we described novel behavioral tasks in detail and suggested that they are appropriate for investigating the relationship between cerebellar function and the temporal processing of absolute and relative timing during almost-identical reaching movements (Yamaguchi and Sakurai, 2014a). In the present study we used muscimol, an agonist of GABAAR (A-type gamma aminobutyric acid receptor), and baclofen, an agonist of GABABR (B-type gamma aminobutyric acid receptor), in rats to inactivate the cerebellar cortex during behavioral tasks. We then tested the impact of Crus II inactivation on the rats’ temporal processing of absolute and relative timing. Our data show that the inactivation of a part of the cerebellar cortex, Crus II, disrupts the temporal prediction of the absolute duration of intervals between events, whereas the relative timing of sequential responses was not affected. These results offer considerable evidence of the characteristics of cerebellar temporal processing and the contribution of the cerebellum to cognitive functions.

## Materials and Methods

### Subjects

Eight male Wistar albino rats (Shimizu Laboratory Supplies, Japan) were used in the experiments. All rats were provided lab chow (1–3 h after daily training sessions) in quantities sufficient to maintain approximately 85–95% of their *ad libitum* weight, except around the time of the surgery. They were allowed free access to water along with daily light exposure between 08:00 and 21:00 except during experimental periods. All experiments were conducted between 10:00 and 20:00. The experiments were conducted in accordance with the *Guidelines for Care and Use of Laboratory Animals at Kyoto University* (2007), with approval from the Animal Research Committee of Kyoto University.

### Apparatus

**FIGURE 1:**
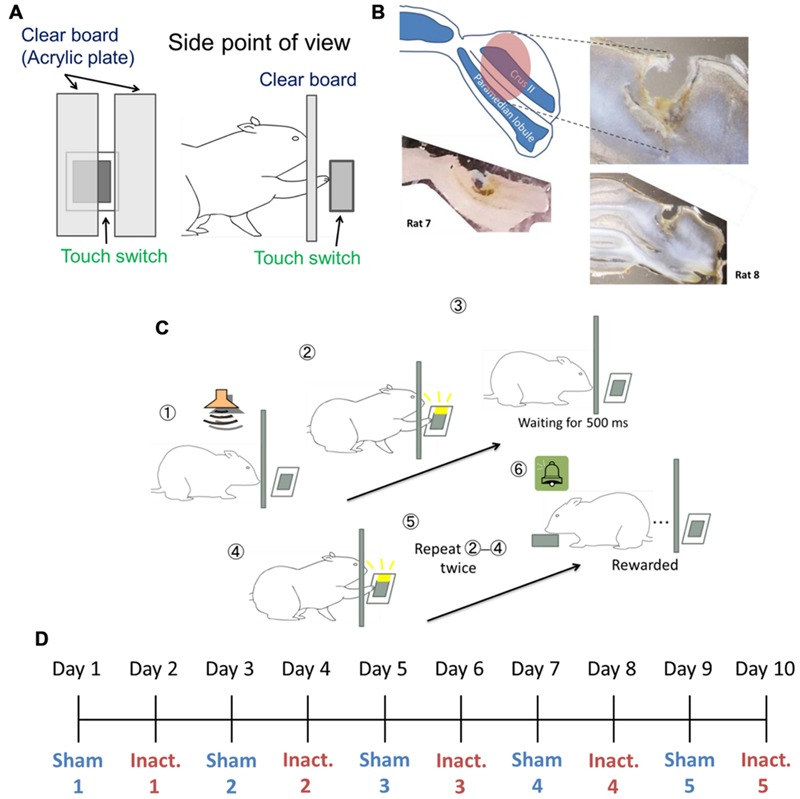
**Experimental setup. (A)** Setting of the touch switch and acrylic plate in the operant chamber. **(B)** Image of the injected area marked by fluorescent dye-conjugated muscimol (see Pharmacological Inactivation). The slices from Rat 7 (bottom left) and Rat 8 (right) were 200 and 150 μm thick, respectively. **(C)** The sequence for the behavioral task. **(D)** Example of the order of sham and inactivation conditions in the test sessions.

The rats were trained for behavioral tasks in an operant chamber measuring 22 cm × 32 cm × 45 cm (Ohara Ika, Japan). One chamber wall had a capacitance touch switch (14 mm × 15 mm) in the center of the wall and 55 mm above the floor, to detect the rats’ behavioral touch responses. When the switch detected a touch, an LED attached to the switch lit up. To cover the switch during inter-trial intervals (ITIs), a guillotine door was used. A clear acrylic plate was inserted between the rats and the switch. There was a 10 mm-wid slit on the right side of the plate, 10 mm away from the center of the plate, and the plate was 10 mm away from the switch. In this way, the rats could touch the switch through the slit using their right paw, but could not touch it with their noses (**Figure 1A**). From behind the opposite chamber wall, a food dispenser delivered 25 mg of food pellets to a food magazine in the center of the wall, 10 mm above the floor. A brief tone sounded every time the dispenser delivered a pellet. The apparatus was controlled using a microcontroller (Arduino Mega 2560, Arduino Software, Italy).

### Implantation Surgery

Under 1–3% isoflurane anesthesia, burr holes were drilled in the skull for the implantation of a stainless guide cannula (26G, Plastics One, USA) into the Crus II of the right cerebellar cortex (–13.08 mm from the bregma and 3.5 mm from the midline). The lateral part of the cerebellar hemisphere is connected to the cerebral cortex and is called “*cerebrocerebellum*” (Lisberger and Thach, 2012). Crus II is involved in motor timing and was investigated in previous studies that assessed the dynamics of the Purkinje cells that receive timing signals from the inferior olive (Welsh et al., 1995; Wise et al., 2010). Therefore, we targeted the lateral part of the Crus II for the purpose of this study. The depth of the guide cannula tips was 3.2 mm from the skull surface. The cannula was attached to the skull with small steel screws and dental cement. To prevent overflowing of blood, the guide cannula was filled in a dummy cannula and covered with a dust cap (Plastics One, USA). After surgery, the rats were allowed to recover for at least 3 days.

### Behavioral Tasks

We used a “duration-based timing task” and a “beat-based timing task” (Yamaguchi and Sakurai, 2014a) in our experiments. In brief, the tasks required the rats to repeatedly touch the switch with their paws at regular intervals of a few hundred milliseconds. When the rats failed to wait for a fixed interval between behavioral responses, an error tone was presented and the guillotine door prevented the rats from touching the switch for 3 s, after which they had to start again from the beginning. To successfully complete the trials, the rats needed to correctly perceive the intervals between touch responses. The duration-based timing task required only one interval to complete the trial, in other words this task involved absolute timing, whereas the beat-based timing task required multiple intervals, and involved relative timing. We combined fixed ratio (FR) and differential reinforcement of low rates of responding (DRL) schedule in these tasks. In the reinforcement schedule, ‘FR *n*’ requires *n* responses to get one reward. This schedule makes animals respond at a high frequency (Reynolds, 1975). ‘DRL *n* ms’ requires *n* ms inter-response time (IRT) between the responses and no response during the interval. Originally, this schedule was used to reduce response frequency (Reynolds, 1975); however, in this study, we used it to make the rats focus on the fixed interval. If the task required *x* responses at intervals of *y* ms to get a reward, the reinforcement schedule was designated “FR *x* DRL *y* ms.” For example, in the FR 3 DRL 500 ms schedule (**Figure 1C**), after the presentation of the trial start tone, the rat could freely touch the switch. Then, the rat had to wait for 500 ms without responding. After 500 ms passed, the rat could touch the switch again. When it repeated this cycle one more time, the rat got a reward. We used FR 2 DRL 500 ms as the absolute timing task and FR 3 DRL 500 ms as the relative timing task.

### Pharmacological Inactivation

To inactivate cerebellar cortex activity, muscimol (an agonist of GABAAR) and baclofen (an agonist of GABABR) were used in combination. The injected liquid solution was prepared from a 20 μl water solution of muscimol hydrobromide (2 mg/200 μl; Sigma–Aldrich Japan), a 20 μl water solution of baclofen hydrochloride (2 mg/ 200 μl; Sigma–Aldrich Japan), and 960 μl saline. The concentration of these drugs was determined based on a pilot experiment. In the pilot experiment, the concentration of the drugs was decreased to a level at which it did not cause ataxic movements (numbness in the limb and/or freezing). This solution, contained in a microliter syringe (Hamilton, USA), was administered to the rats’ brains while they were awake, via the cerebellar guide cannula, through an internal cannula connected by a polyethylene tube (Plastics One, USA). A total of 0.5 μl was injected at a rate of 30 μl/h prior to each session of inactivation conditions. The injections were controlled by an automatic syringe pump (Fusion Touch 100, ISIS, Japan).

To identify the area through which the solution spread, we injected fluorescent dye-conjugated muscimol (M-23400; Molecular Probes, USA) 3 h before conducting a histological procedure. We sectioned the brains of Rat 7 and Rat 8 at 200 or 150 μm intervals, respectively. The solution was prepared from a 40 μl water solution of fluorescent dye-conjugated muscimol (2 mg/200 μl) and 960 μl of saline. The procedure for injection was the same as during the test sessions. These histological examinations showed that the solution diffused approximately 2 mm^3^ from the center of the injected site (**Figure 1B**).

### Training and Experimental Design

At the beginning of training, rats received a pellet after touching the switch in the absence of the acrylic plate. Later on, a continuous reinforcement schedule was achieved using the acrylic plate to allow the rats to touch the switch only with their right paws. The cannula implantation surgeries were conducted after the rats had learned how to touch the switch. After surgery and more than 3 days of rest, the rats were exposed to FR 2 DRL 500 ms schedule training. In the training and test sessions, a tone was presented at the start of the session and after each ITI. We defined a trial as the time from tone presentation to reward presentation. The “correct rate” represented the proportion of trials in which there were no erroneous responses from the tone presentation to the reinforcement. This property was used as a learning criterion in training and during behavioral analyses under experimental conditions. Each session took 1 h and each rat underwent one session per day. In the training sessions, we judged the rats to have learned the schedule when their performance criteria were achieved within one session. The criteria were that they had to achieved correct rates of ≥70% and that ≥80% of IRTs had to be <1500 ms. When the rats could attain these criteria in three of four consecutive sessions, they advanced to the test sessions. The test sessions were immediately preceded by one of two types of injection: the inactivation injection described in Section “Pharmacological Inactivation” and a sham (control), in which the injected solution was saline. These sessions were alternated and repeated five times each for each rat (**Figure 1D**). After completing a total of 10 sessions, the rats were given the FR 3 DRL 500 ms training. We used different criteria to evaluate performance under this schedule: ≤30% incorrect IRTs for the session and ≥80% IRTs < 1500 ms. Again, when the rats met these criteria in three of four consecutive sessions, they underwent the test sessions under the FR 3 DRL 500 ms schedule, with injection types following the same procedure as before.

### Histology

Procedures were almost identical to those used in previous studies (Sakurai and Takahashi, 2013; Terada et al., 2013). After the rats had undergone the FR 3 DRL 500 ms tests, they were euthanized with an overdose of the anesthetic sodium pentobarbital (120 mg/kg). Their brains were extracted, perfused and fixed with a 10% buffered for-malin solution. They were then sectioned at 80 μm intervals, after which the location of the cannula tips and tracks in the brains were identified with the aid of a stereotaxic atlas (Paxinos and Watson, 2009).

### Behavioral Data Analysis

Inter-response times, correct rates, proportion of incorrect IRTs, reward counts (i.e., number of trials), and the rats’ weights during each session were recorded and analyzed. IRTs of 0–100 ms were excluded from analysis because these responses may have been rapid multiple involuntary responses or response bursts. To avoid the warm-up effect, the first three trials in each session were excluded from the analysis. We excluded sessions in which the rat’s performance was very different because of changes in other conditions (e.g., considerable increase in body weight, or malfunction of the equipment). In these cases we repeated the sessions until the rats completed comparable 10 sessions. In the present task, IRTs longer than 500 ms were regarded as correct responses irrespective of how long they were. To exclude the impact of extremely long IRTs, we categorized the IRTs by 50 ms bins, and IRTs longer than 3000 ms were combined into a “≥3000 ms” response class. We used a two-sided Kolmogorov–Smirnov (K–S) test, which is suitable for the analysis of categorical data, to compare representative values and/or IRT distributions between the two conditions. Even when the K–S test detected significant differences between two conditions, it does not determine which specific parameter, whether the representative value or the shape of distribution, caused the difference. We then used the two-sided Mood test to confirm statistically difference of the shapes of the IRT distributions. One-sided Wilcoxon signed rank test was used to compare averages of correct rates, proportions of incorrect IRTs, reward counts and rats’ weight between two conditions.

## Results

Our histological examination has confirmed that the cannulas were inserted into same targeted areas in all eight rats and that the injections of the inactivation and sham solutions, in Rat 7 and 8, reached the target areas, namely, the Crus II and paramedian lobule, which are involved in arm and paw movements (Apps and Hawkes, 2009; see Histology, **Figure 1B**).

### Temporal Prediction of Absolute Duration Under Crus II Inactivation

We compared differences in IRT distribution, averages of correct rates, proportions of incorrect IRTs (i.e. IRTs of less than 500 ms), reward counts and rats’ body weights (see Behavioral Data Analysis) between the sham and inactivation conditions under the FR 2 DRL 500 ms schedule (**Table 1**). The average reward count in the inactivation condition was not significantly lower than that in the sham condition in any of the rats. This shows that the rats had little motor dysfunction or depressed motivation as a result of Crus II inactivation. There was no difference in body weights between the sham and inactivation conditions in any of the rats (**Table 1**), indicating that behavioral performance could not have been affected by motivation or body condition, since body condition did not change. The K–S test, however, revealed significant overall differences between the sham and inactivation conditions in seven of the eight rats (**Table 1**, orange). We therefore performed further analysis on only the data from the seven rats in which there was a difference. The Mood test revealed significant differences between the two conditions in three of these rats (**Table 1**, orange). Thus the means and/or modes of the IRT distribution was significantly affected by inactivation of part of the cerebellar cortex in four of the eight rats and the shape of the IRT distribution was significantly affected by the inactivation in three of the eight rats (see Behavioral Data Analysis). This suggests that the cerebellar cortex has strong effects on the processing of absolute timing of voluntary movements.

**Table 1:** Comparison of test parameters for each rat between the sham and inactivation conditions in the FR 2 DRL 500 ms tests.

	FR 2 DRL 500 ms		Sham × inactivation		
	K–S test Mood test)	Correct rate	Proportion of incorrect IRTs	Reward counts	Body weight
Rat 1	***p* = 0.01004^∗^** (*p* = 0.1815)	Sham: 86.8 ± 9.3 Inactivation: 84.6 ± 8.1 *p* = 0.3125	Sham: 0.16 ± 0.13 Inactivation: 0.182 ± 0.1 *p* = 0.3125	Sham: 106.8 ± 17.6 Inactivation: 99.8 ± 23.9 *p* = 0.5	Sham: 477 ± 0.9 Inactivation: 476 ± 4.0 *p* = 0.3422
Rat 2	*p* = 0.1377	Sham: 74.8 ± 9.6 Inactivation: 74.3 ± 8.3 *p* = 0.5	Sham: 0.249 ± 0.1 Inactivation: 0.244 ± 0.1 *p* = 0.3125	bbb**Sham: 145.8 ± 14.6** Inactivation: **161.8 ± 21.2** ***p* = 0.03125^∗^**	Sham: 444 ± 0.7 Inactivation: 446 ± 1.5 *p* = 0.1807
Rat 3	***p* = 5.52E-04^∗∗^** (*p* = 0.8994)	ccc**Sham: 73.6 ± 2.5** Inactivation**: 66.2 ± 2.1** ***p* = 0.03125^∗^**	bbb**Sham: 0.253 ± 0.03** Inactivation**: 0.303 ± 0.01** ***p* = 0.03125^∗^**	Sham: 166.8 ± 12.5 Inactivation: 160.4 ± 12.2 *p* = 0.2491	Sham: 440 ± 0.7 Inactivation: 439 ± 1.3 *p* = 0.2931
Rat 4	***p* = 0.006262^∗∗^** (*p* = 0.1027)	Sham: 81.4 ± 4.11 Inactivation: 75.3 ± 6.1 *p* = 0.09375	Sham: 0.177 ± 0.04 Inactivation: 0.232 ± 0.05 *p* = 0.1562	Sham: 205.2 ± 18.9 Inactivation: 218.6 ± 12.8 *p* = 0.2188	Sham: 480 ± 3.4 Inactivation: 479 ± 4.9 *p* = 0.3422
Rat 5	***p* = 0.008902^∗∗^** (***p* = 0.004468^∗∗^**)	Sham: 89.6 ± 0.95 Inactivation: 89.6 ± 5.72 *p* = 0.5	Sham: 0.104 ± 0.01 Inactivation: 0.11 ± 0.07 *p* = 0.4062	Sham: 185.2 ± 34.8 Inactivation:188.6 ± 17.0 *p* = 0.5	Sham: 438 ± 7.1 Inactivation: 438 ± 2.2 *p* = 0.4276
Rat 6	***p* = 0.005926^∗∗^** (***p* = 0.008517^∗∗^**)	Sham:84.4 ± 7.54 Inactivation:80.7 ± 9.51 *p* = 0.1562	Sham: 0.155 ± 0.08 Inactivation: 0.212 ± 0.14 *p* = 0.1562	Sham:198.0 ± 46.3 Inactivation:207.6 ± 18.4 *p* = 0.4062	sham: 439 ± 3.9 Inactivation: 439 ± 5.6 *p* = 0.4459
Rat 7	***p* = 0.008089^∗∗^** (*p* = 0.4325)	Sham:92.2 ± 2.86 Inactivation:92.8 ± 2.96 *p* = 0.4062	Sham: 0.083 ± 0.03 Inactivation: 0.068 ± 0.03 *p* = 0.4062	Sham:.128.8 ± 6.3 Inactivation:145.4 ± 41.8 *p* = 0.1562	Sham: 507 ± 3.1 Inactivation: 510 ± 2.8 *p* = 0.2113
Rat 8	***p* = 0.004028^∗∗^** (***p* = 1.606E-06^∗∗∗^**)	Sham: 73.1 ± 2.6 Inactivation:69.1 ± 7.9 *p* = 0.1562	Sham: 0.297 ± 0.03 Inactivation: 0.354 ± 0.08 *p* = 0.09375	Sham: 141.0 ± 20.0 Inactivation: 148.6 ± 19.5 *p* = 0.2919	Sham: 490 ± 1.6 Inactivation: 490 ± 1.5 *p* = 0.5

*K–S test: Kolmogorov–Smirnov test; Data presented are mean ± SD; ^∗^*p <* 0.05, ^∗∗^*p <* 0.01, ^∗∗∗^*p <* 0.001; Colored text highlights statistically significant results (red = sham > inactivation, blue = inactivation > sham, orange = two-sided test).*

**FIGURE 2:**
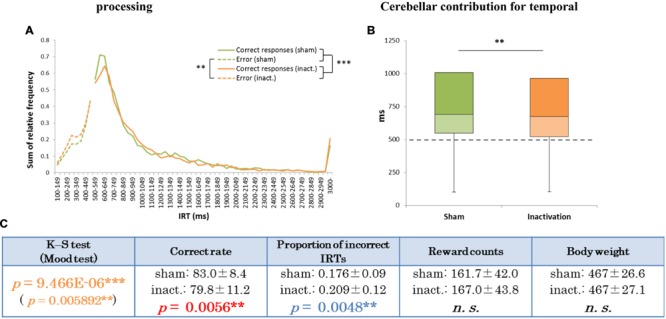
**Behavioral performance of all seven rats that showed inactivation effects in the FR 2 DRL 500 ms tests. (A)** IRT distributions. “Correct responses” represents IRTs that were longer than 500 ms, whereas “error” represents IRTs that were shorter than 500 ms. **(B)** Box plot of the IRT distributions for the seven rats in the sham and inactivation conditions. The dotted line indicates the cut-off (500 ms). **(C)** Test parameters for the seven rats combined. Data presented are mean ± SD. Colored *p*-values highlight significant differences as in **Table 1**. ^∗∗^*p <* 0.01, ^∗∗∗^*p <* 0.001.

Analysis of the data for all rats combined revealed significant differences in IRT distributions (K–S and Mood tests) between the sham and inactivation conditions (**Figure 2**). Their average correct rates were also significantly lower and proportions of incorrect IRTs significantly higher in the inactivation condition than in the sham condition (**Figure 2B**). The combined IRT distributions revealed a decrease in frequency of the shortest correct IRTs and an increase in frequency of incorrect IRTs under inactivation (**Figure 2A**).

**FIGURE 3:**
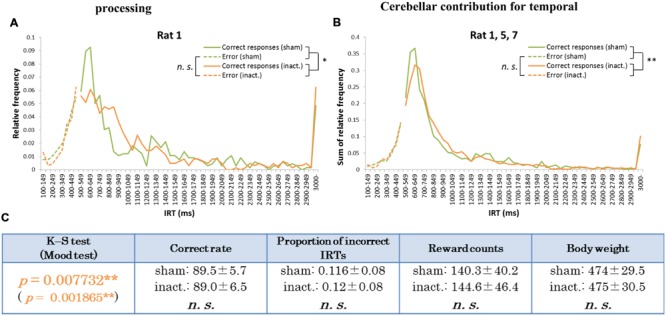
**Behavioral performance of the longer decline rats in the FR 2 DRL 500 ms tests. (A)** IRT distributions for Rat 1 in the sham and inactivation conditions. “Correct responses” and “error” as for **Figure 2**. **(B)** IRT distributions for Rat 1, 5, and 7 combined. **(C)** Parameters of the longer decline rats considered as a group. Data presented are mean ± SD. Orange *p*-values highlight significant differences. ^∗^*p <* 0.05, ^∗∗^*p <* 0.01.

Histogram plots of the IRT distributions of each rat revealed two types of frequency declines in IRT from the sham to the inactivation condition. We called the first type the “longer decline,” in which the frequency of the shortest correct IRT decreased and that of longer IRTs increased in the inactivation condition (**Figures 3A,B**). For example, for Rat 1 (**Figure 3A**), in the sham condition the IRT distribution increased sharply for IRTs of 500–600 ms and decreased sharply as they reached 700 ms. However, under the inactivation condition, the frequencies of IRTs of 500–900 ms were similar, and only decreased for IRTs > 900 ms. Rat 5 and 7 showed the same tendency. Statistical test results of the combined performance data for these three subjects treated as a group and comparison of parameters between the sham and inactivation conditions were similar to those of Rat 1 (**Table 1**), except for the Mood test result (**Figures 3B,C**). We designated these subjects as the “longer decline rats,” and concluded that for these individuals temporal prediction of absolute duration became longer than learned intervals, with little effect on motor ability, following inactivation of the cerebellar cortex.

**FIGURE 4:**
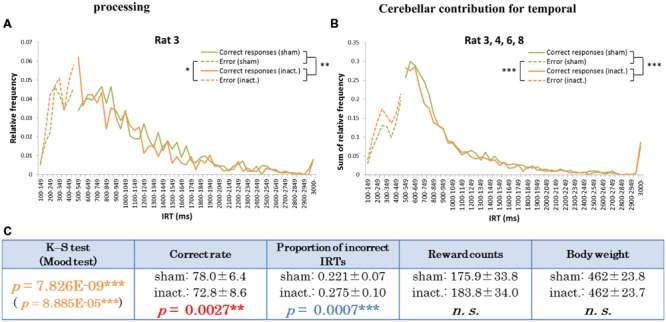
**Behavioral performance of the shorter decline rats in the FR 2 DRL 500 ms tests. (A)** IRT distributions for Rat 3 in the sham and inactivation conditions. “Correct responses” and “error” as for **Figure 2**. **(B)** IRT distributions for Rat 3, 4, 6, and 8 combined. **(C)** Parameters of the shorter decline rats as a group. Data presented are mean ± SD. Colored *p*-values highlight significant differences as in **Table 1**. ^∗^*p <* 0.05, ^∗∗^*p <* 0.01, ^∗∗∗^*p <* 0.001.

The other type of IRT frequency decline was the “shorter decline.” For Rat 3, for example, average correct rates were significantly lower and proportions of incorrect IRTs were significantly higher in the inactivation condition than in the sham condition (**Table 1**). The IRT distribution for this individual in the inactivation condition showed a sharp increase for IRTs of 300–500 ms and then decreased again. Under the sham condition, however, IRT frequencies gradually increased for IRTs 500–900 ms (**Figure 4A**). Rat 3 was the only subject that showed significant differences in correct rates and proportion of incorrect IRTs between the sham and inactivation conditions. The results for Rats 4, 6, and 8 did, however, show some similarities to those for Rat 3, viz., relatively larger differences in correct rates (at least 4%) and proportion of incorrect IRTs (at least 0.5) than those seen in the longer decline rats, which had maximum differences of 1.2% in correct rates and 0.2 in the proportion of incorrect IRTs (**Table 1**). Furthermore, Rats 4, 6, and 8 showed declines in the IRT distribution for IRTs from 700 to 900 ms in the inactivation condition, similar to those shown by Rat 3 (**Figure 4B**). We therefore regarded Rats 3, 4, 6, and 8 as “shorter decline rats.” Analysis of the combined data for this group revealed significant differences in their IRT distributions (K–S and Mood tests); also, their average correct rates were significantly lower and proportions of incorrect IRTs were significantly higher in the inactivation condition than those in the sham condition (**Figure 4C**). From this we concluded that for those rats, temporal prediction of absolute duration became shorter than learned intervals, also with little effect on motor ability, following inactivation of the cerebellar cortex.

To summarize the results of the FR 2 DRL 500 ms tests (the absolute timing task), inactivation of part of the cerebellar cortex generally decreased the accuracy of temporal prediction. However, the timing changed in both directions, i.e., either longer or shorter than the originally learned time, depending on the individual.

### Temporal Prediction of Relative Timing Under Crus II Inactivation

Rat 8 was excluded from the FR 3 DRL 500 ms tests because it did not achieve the learning criteria and eventually stopped touching the switch altogether. We therefore analyzed only the data from the other seven rats in regard to the relative timing task.

As for the FR 2 DRL 500 ms test, we compared differences in IRT distributions, average correct rates, proportions of incorrect IRTs, reward counts, and the rats’ body weights between the sham and inactivation conditions (**Table 2**). In the FR 3 DRL 500 ms tests, there was no difference in average reward count or body weight between the sham and inactivation conditions in any of the rats. This indicates that behavior was not affected by body condition (since it did not change) and that motor function and motivation were not affected by Crus II inactivation. The K–S and Wilcoxon signed rank tests revealed no significant differences between the sham and inactivation conditions in any of the parameters for any of the rats (**Table 2**).

**Table 2:** Comparison of test parameters for each rat between the sham and inactivation conditions in the FR 3 DRL 500 ms tests.

	FR 3 DRL 500ms sham × inactivation				
	K–S test (Mood test)	Correct rate	Proportion of incorrect IRTs	Reward counts	Body weight
Rat 1	*p* = 0.3287	Sham: 90.5 ± 7.0 Inactivation: 90.9 ± 4.4 *p* = 0.5	Sham: 0.05 ± 0.04 Inactivation: 0.05 ± 0.02 *p* = 0.5	Sham: 89.4 ± 15.9 Inactivation: 84.2 ± 44.7 *p* = 0.5	Sham: 478 ± 2.8 Inactivation: 477 ± 2.4 *p* = 0.1393
Rat 2	*p* = 0.3312	Sham: 87.3 ± 4.7 Inactivation: 86.4 ± 3.0 *p* = 0.4062	Sham: 0.06 ± 0.03 Inactivation: 0.08 ± 0.03 *p* = 0.3125	Sham: 83.2 ± 13.7 Inactivation: 100.8 ± 20.7 *p* = 0.0625	Sham: 461 ± 1.8 Inactivation: 462 ± 1.1 *p* = 0.3946
Rat 3	*p* = 0.1739	Sham: 68.4 ± 6.6 Inactivation: 73.6 ± 4.6 *p* = 0.0625	Sham: 0.185 ± 0.03 Inactivation: 0.158 ± 0.04 *p* = 0.1562	Sham: 131.6 ± 15.0 Inactivation: 146.0 ± 5.0 *p* = 0.09375	Sham: 442 ± 0.9 Inactivation: 442 ± 1.7 *p* = 0.2931
Rat 4	*p* = 0.08083	Sham: 86.3 ± 2.1 Inactivation: 86.8 ± 3.0 *p* = 0.4062	Sham: 0.074 ± 0.02 Inactivation: 0.067 ± 0.02 *p* = 0.3125	Sham: 160.0 ± 50.8 Inactivation: 186.4 ± 19.1 *p* = 0.2919	Sham: 472 ± 3.4 Inactivation: 472 ± 2.7 *p* = 0.4062
Rat 5	*p* = 0.2928	Sham: 80.9 ± 4.9 Inactivation: 84.8 ± 2.4 *p* = 0.1562	Sham: 0.103 ± 0.02 Inactivation: 0.085 ± 0.02 *p* = 0.2188	Sham: 134.8 ± 44.9 Inactivation: 148.6 ± 18.6 *p* = 0.5	Sham: 416 ± 8.3 Inactivation: 418 ± 3.6 *p* = 0.2082
Rat 6	*p* = 0.1116	Sham: 82.8 ± 7.9 Inactivation: 80.0 ± 13.1 *p* = 0.4062	Sham: 0.114 ± 0.06 Inactivation: 0.132 ± 0.10 *p* = 0.3125	Sham: 181.2 ± 26.9 Inactivation: 199.6 ± 12.8 *p* = 0.09375	Sham: 435 ± 3.6 Inactivation: 434 ± 3.3 *p* = 0.09873
Rat 7	*p* = 0.8156	Sham: 92.7 ± 2.0 Inactivation: 93.2 ± 4.8 *p* = 0.3932	Sham: 0.04 ± 0.01 Inactivation: 0.04 ± 0.03 *p* = 0.5	Sham: 132.6 ± 16.9 Inactivation: 131.8 ± 36.4 *p* = 0.5	Sham: 490 ± 3.1 Inactivation: 491 ± 0.8 *p* = 0.427

*K–S test: Kolmogorov–Smirnov test; Data presented are mean ± SD.*

**FIGURE 5:**
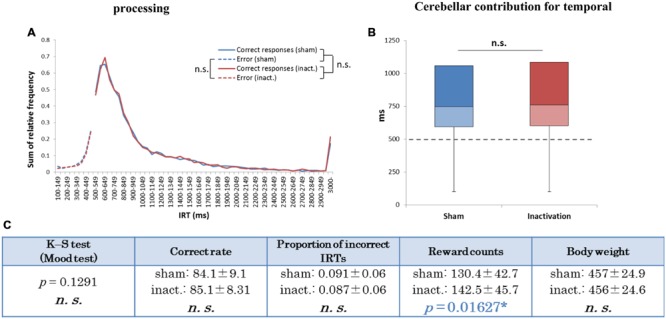
**Behavioral performance of all seven rats that performed the test successfully in the FR 3 DRL 500 ms tests. (A)** IRT distributions. “Correct responses” and “error” as for **Figure 2**. **(B)** Box plot of the IRT distributions for the seven rats in the sham and inactivation conditions. The dotted line indicates the cut-off (500 ms). **(C)** Test parameters for the seven rats combined. Data presented are mean ± SD. The blue *p*-value highlights a significant difference as for **Table 1** (inactivation > sham). ^∗^*p <* 0.05.

In the group analysis (all seven rats combined), there were no significant differences in IRT distribution, average correct rates, proportions of incorrect IRTs, or body weight between the sham and inactivation conditions (**Figure 5**). The average reward count, however, was significantly greater under inactivation than in the sham condition (**Figure 5C**). This shows that the rats did not experience ataxic movements or depressed motivation as a result of Crus II inactivation. Inactivation of part of the cerebellar cortex did not, therefore, affect the rats’ temporal processing during rhythmic continuous event intervals, implying that their processing of relative timing did not require computation by the whole cerebellar cortex, unlike their processing of absolute timing.

**FIGURE 6:**
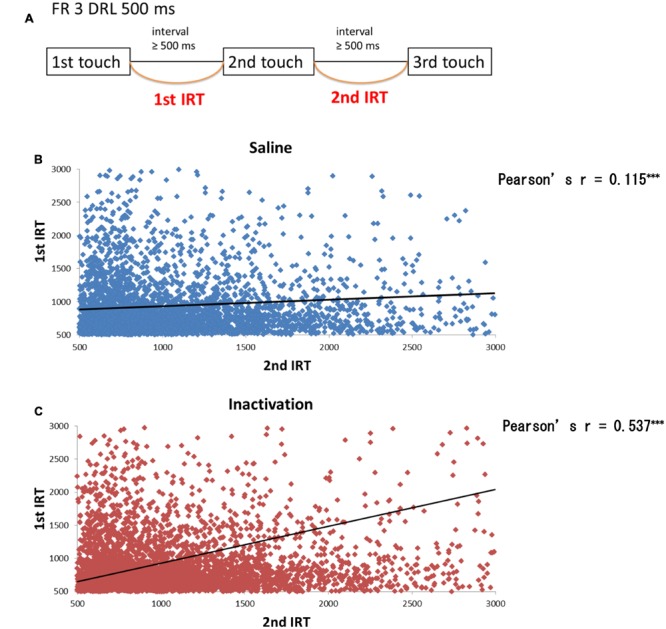
**Correlation between the first and second IRTs of seven rats in the FR 3 DRL 500 ms tests. (A)** A schematic illustration of the behavioral sequence in the FR 3 DRL 500 ms test. **(B)** The scatter plot of the first and second IRTs of the seven rats in the sham condition. There was a significant correlation between the first and second IRTs (*p <* 0.001, paired *t*-test). The IRTs that were > 3000 ms, regarded as the outliers, were excluded from this figure but not from the statistical analysis. The solid line indicates an approximate curve. **(C)** The scatter plot of the first and second IRTs of the seven rats in the inactivation condition. There was a significant correlation between the first and second IRTs (*p <* 0.001, paired *t*-test). ^∗∗∗^*p <* 0.001.

We further analyzed the results of the FR 3 DRL 500 ms tests by separating the IRT distributions into IRTs between the first and second touch responses (first IRTs) and into those between the second and third responses (second IRTs; **Figure 6A**). If the relative timing task required beat-based, rhythmic movements, the first and second IRTs in individual trials should correlate with each other. The paired *t*-test revealed that there were significant correlations between the two IRTs both in the sham and inactivation conditions (**Figures 6B,C**). The two IRTs positively correlated in both conditions (Pearson’s *r*: sham, 0.115; inactivation, 0.537).

**FIGURE 7:**
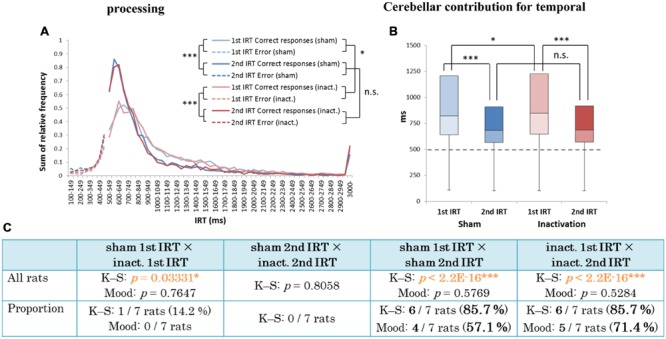
**Behavioral performance of all seven rats that performed the test successfully in the FR 3 DRL 500 ms tests. (A)** IRT distributions. “Correct responses” and “error” as for **Figure 2**. **(B)** Box plot of the IRT distributions for the seven rats in the sham and inactivation conditions. The dotted line indicates the cut-off (500 ms). **(C)** Results of the K–S and Mood tests of differences between distributions of first and second IRTs within the sham and inactivation conditions, and within these two categories between the two conditions. (Top) Orange *p*-values highlight significant differences. ^∗^*p <* 0.05, ^∗∗∗^*p <* 0.001. (Bottom) The numbers (and percentages) of subjects that showed significant differences in the K–S and Mood tests.

There were significant differences between the first IRT distributions in the sham and inactivation conditions and between the first and second IRT distributions within each condition (K–S test; **Figure 7C**). In the individual analysis, six of the seven rats showed significant differences between their first and second IRT distributions within both conditions, whereas only one of the seven rats showed significant differences in its first IRT distribution between the two conditions (**Figure 7C**). The Mood test showed significant differences between the first and second IRT distributions for five rats within the sham condition and six within the inactivation condition. However, no subjects showed significant differences between the two conditions for either their first or second IRT distributions (**Figure 7C**). Therefore, we concluded that Crus II inactivation may have minimal effects on rats’ performances with respect to relative timing. In all subjects, the second IRTs were closer to the DRL value (500 ms) than the 1st IRTs in both the sham and inactivation conditions (**Figures 7A,B**). This pattern was also found in our previous study that utilized this schedule (Yamaguchi and Sakurai, 2014a) and was denominated by us the “order effect.” Inactivation of the cerebellar cortex did not change performance in the FR 3 DRL 500 ms tests, even when we examined the IRT distributions for first and second IRTs separately; This provides support for the concept that the cerebellar cortex is not necessary for processing relative timings.

## Discussion

In the present study we tested the impact of Crus II inactivation on temporal processing of absolute and relative timing, using two different tests: FR 2 DRL 500 ms and FR 3 DRL 500 ms. IRT distributions in the FR 2 DRL 500 ms test changed significantly under inactivation of the cerebellar cortex (**Figure 2**). However, in the FR 3 DRL 500 ms test, IRT distributions were not affected by the inactivation in any of the rats (**Figure 5**). This suggests that inactivation of the cerebellar cortex disrupted accurate temporal prediction in absolute timing tasks, but did not affect the performance of relative timing tasks. Although the tasks involved not only temporal prediction but also motor function, the disruption of the temporal prediction was probably crucial for the decline in the absolute timing task rather than the ataxic movements due to the Crus II inactivation. This conclusion is supported by two observations. (1) Average reward counts were not affected by cerebellar inactivation in any of the rats. If the motor function had been significantly disrupted, the rats could not have completed a comparable number of trials in the same amount of time as in the sham condition. In fact, the rats showed no abnormal movements, although we observed that some rats showed ataxic movements in the pilot study in which the concentration of the drugs was higher than in this study (cf. Pharmacological Inactivation). (2) If only the motor functions had been disrupted, the subjects could have adjusted the timing of their movements using their intact temporal function. However, it cannot be concluded that the rats did not have any disruption in their movements without a detailed analysis of their kinematics. We, therefore, conclude that a part of the cerebellar cortex, Crus II, is involved in the accurate temporal prediction of absolute timing and not purely in motor function.

In the results from the absolute timing task (i.e., the FR 2 DRL 500 ms test), we found two distinct patterns in the decline of accuracy in prediction of the learned interval. In **Figure 3**, the proportions of the IRTs of 500–700 ms in the sham and inactivation conditions were 39 and 36%, respectively, in the combined distributions (**Figure 3B**) and 29 and 22%, respectively, in the distribution of Rat 1 (**Figure 3A**). This weakening of the inactivation effect might be caused by the difference in the efficacy of the Crus II inactivation in those three rats, although there were significant differences between the sham and inactivation conditions in those three rats. The effect of the Crus II inactivation in Rat 5 and 7 might be weaker than that in Rat 1. In previous studies which ablated or inactivated a part of the cerebellum, declines in the accuracy of prediction of learned intervals were also observed (Welsh and Harvey, 1989; Bao et al., 2002; Koekkoek et al., 2003; Koch et al., 2007; Lee et al., 2007; Hoﬄand et al., 2012). One study on transgenic mice in which parallel fiber-Purkinje cell LTD was impaired demonstrated that long-term depression of the Purkinje cells did not affect the time of conditioned eyelid responses (Koekkoek et al., 2003); furthermore, the responses were consistently earlier than in the wild type. Similarly, Hoﬄand et al. (2012) showed that the timing of conditioned eyelid responses became earlier when theta burst stimulations were applied to the right cerebellar hemisphere of human subjects. In contrast, in experiments using classical eyelid conditioning, either ablation or reversal inactivation of the deep cerebellar nuclei (interpositus nuclei) caused delayed eyelid responses (Welsh and Harvey, 1989; Bao et al., 2002). Other human studies showed that participants overestimated millisecond intervals when their cerebella were inactivated by repetitive transcranial magnetic stimulation (Koch et al., 2007; Lee et al., 2007). Thus, declines in timing accuracy have been reported in several studies. The patterns of decline in accuracy of IRTs observed in the present study may be explained by variation in the response timings of rats. Average correct rates and proportions of incorrect IRTs in the longer decline group were higher and lower, respectively, than those in the shorter decline rats (**Figures 3C** and **4C**). We can therefore speculate that the longer decline rats managed to identify their timings earlier and shorter decline individuals tended to be slower when they could not respond at the correct time. For this reason, the cerebellar cortex may modulate in either direction to attempt to achieve accurate timing to act.

Finally, Crus II inactivation did not affect the rats’ performance in the relative timing tasks. Under the FR 3 DRL 500 ms schedule, there were significant differences in the reward counts between the sham and inactivation conditions (**Figure 5C**). However, significant differences were not found among all the individual rats, and individual variation was high in the reward counts (**Table 2**). The significant differences were probably caused by the increasing number of observations, although the inactivation of Crus II may have made the animals more efficient. This is consistent with previous studies that showed that impairment of the cerebellum in human subjects disrupted the accuracy of the timing of discontinuous but not of continuous events (Grube et al., 2010). However, Ohmae et al. (2013) contradicted Grube et al. (2010) and suggested that timing of continuous sensory inputs relates to the cerebellum, because in their experiments deep cerebellar nucleus neurons responded to repetitive isochronous sensory cues. In the task used in the study of Ohmae et al. (2013), it is possible that the activity of deep cerebellar nucleus neurons reflected the predicted absolute durations because the intervals between repetitive stimuli were fixed within each trial and the neural activity increased as the trials continued. We therefore conclude that reflection of absolute duration or sensory responses to rhythmic stimuli is a reasonable explanation for our findings. A comparison of cerebellar neuronal activities between absolute and relative timing tasks is required to answer this question more directly. Although the cerebellar obligate role for temporal processing may be in the prediction of absolute and not of relative timing, it may be necessary to demonstrate differences in cerebellar neuronal activities between the two types of timing to elucidate this.

## Author Contributions

KY and YS designed the research; KY performed the research and analyzed the data; and KY and YS wrote the paper.

## Conflict of Interest Statement

The authors declare that the research was conducted in the absence of any commercial or financial relationships that could be construed as a potential conflict of interest.
